# Challenges in the Implementation of a Mobile Application in Clinical Practice: Case Study in the Context of an Application that Manages the Daily Interventions of Nurses

**DOI:** 10.2196/mhealth.2344

**Published:** 2013-06-12

**Authors:** Frederic Ehrler, Rolf Wipfli, Douglas Teodoro, Everlyne Sarrey, Magali Walesa, Christian Lovis

**Affiliations:** ^1^Division of Medical Information SciencesUniversity Hospitals of GenevaGenevaSwitzerland; ^2^University of GenevaGenevaSwitzerland; ^3^Direction of NursingUniversity Hospitals of GenevaGenevaSwitzerland

**Keywords:** hospital information systems, computers, handheld, equipment design, nurses, mobile health, pilot projects, user-computer interface

## Abstract

**Background:**

Working in a clinical environment requires unfettered mobility. This is especially true for nurses who are always on the move providing patients’ care in different locations. Since the introduction of clinical information systems in hospitals, this mobility has often been considered hampered by interactions with computers. The popularity of personal mobile assistants such as smartphones makes it possible to gain easy access to clinical data anywhere.

**Objective:**

To identify the challenges involved in the deployment of clinical applications on handheld devices and to share our solutions to these problems.

**Methods:**

A team of experts underwent an iterative development process of a mobile application prototype that aimed to improve the mobility of nurses during their daily clinical activities. Through the process, challenges inherent to mobile platforms have emerged. These issues have been classified, focusing on factors related to ensuring information safety and quality, as well as pleasant and efficient user experiences.

**Results:**

The team identified five main challenges related to the deployment of clinical mobile applications and presents solutions to overcome each of them: (1) Financial: Equipping every care giver with a new mobile device requires substantial investment that can be lowered if users use their personal device instead, (2) Hardware: The constraints inherent to the clinical environment made us choose the mobile device with the best tradeoff between size and portability, (3) Communication: the connection of the mobile application with any existing clinical information systems (CIS) is insured by a bridge formatting the information appropriately, (4) Security: In order to guarantee the confidentiality and safety of the data, the amount of data stored on the device is minimized, and (5) User interface: The design of our user interface relied on homogeneity, hierarchy, and indexicality principles to prevent an increase in data acquisition errors.

**Conclusions:**

The introduction of nomadic computing often raises enthusiastic reactions from users, but several challenges due to specific constraints of mobile platforms must be overcome. The ease of development of mobile applications and their rapid spread should not overshadow the real challenges of clinical applications and the potential threats for patient safety and the liability of people and organizations using them. For example, careful attention must be given to the overall architecture of the system and to user interfaces. If these precautions are not taken, it can easily lead to unexpected failures such as an increased number of input errors, loss of data, or decreased efficiency.

## Introduction

Hospitals are increasingly using Clinical Information Systems (CIS). The introduction of computers to manage patient information has deeply modified the workflow of the care provider [1]. It has numerous positive effects, such as reduced archiving costs, facilitated administrative tasks, eased access to patient data, structured information, and more generally, improved patient safety through decision support and better access to information.

While dematerialization is one of the major advantages of computerization, it is surprisingly often associated with decreased mobility. This apparent contradiction is well observed in clinical settings with a strong dependence on computers [2]. As long as patient data were kept on paper, care providers could carry and access patient information easily, everywhere in the hospital. With the introduction of CIS, information is no longer stored on physical media. Consequently, caregivers rely on the presence of a computer to access the information.

There is a long history of attempts to provide a ubiquitous access to clinical information. This history begins in 1975 with the first laptop produced by IBM. A new step was reached with the joint appearance of the first PDA and of wireless technology in 1996. Despite a long history, the numerous attempts to provide ubiquitous access to clinical information with these technologies cannot be considered completely successful. The use of wireless networks considerably improves the situation [3], but only to a limited extent. Laptops still require being moved on a trolley, and their autonomy, while improving, is often limited. The other attempts to provide ubiquitous access to clinical information with these technologies have been performed with PDAs. Unfortunately, this technology was not mature enough for the numerous constraints of a medical environment [4]. All these attempts have revealed that using mobile devices to offer ubiquitous computing is a challenging task.

The recent evolution of mobile devices, such as decreased size and cost, better screen resolution, increased computational power, and extended power autonomy, has opened new possibilities to providing strongly integrated mobile tools in health care. Thus, it is now possible to consider having one highly mobile device per care provider, perhaps their personal device, always in their pocket. However, adapting existing applications to these new platforms is a delicate task and requires new models of interactions. History demonstrates that the transition has to be done carefully [5]. Several papers show that mobile devices can lead to increased time for data acquisition, increased errors, and omissions rate [6,7].

This paper presents the major challenges inherent in the deployment of a mobile clinical application. In order to identify these challenges, a group of experts relied on several years of experience with the development of various applications on devices available on the market, such as medical knowledge management on the Palm in the early 2000s and more recently, applications to manage nurse daily interventions on Android and iOS applications for the Geneva community [8,9].

### Mobile Computing for Clinicians

Until recently, most mobile medical applications were developed on Personal Digital Assistants (PDAs) such as Palm platforms. The first generations of PDAs did not have the functionality to manage complete electronic medical records or store large graphics. However, such handheld devices were considered by users as excellent tools for managing clinical information and accessing it at the point of care. Indeed, they were one of the few platforms with an interface supporting input via a stylus, expandable memory, software upgradability, a method of developing custom-built software for the device, and network connectivity [10].

Most applications running on these devices were generic but not directly connected to the clinical information system (CIS) [11]. As early as 2002, Porn and Patrick [12] identified the following health care applications that could be run successfully on a mobile device:

- E-prescription: It allows care providers to access basic patient information and check formulary compliance before writing prescriptions. Potentially harmful events can be detected. Prescriptions can be transmitted directly to a pharmacy. The main benefits are a reduction in medication errors and fewer calls from pharmacies due to illegible handwriting [13].
- Workload capture: The application allows care providers to view schedules, capture patient care, and access or update patient information all at the point of care.
- Order entry: Applications to order certain tests can be scheduled, delivered to a central processing unit, and acted upon. This reduces errors due to misplacement of application forms.
- Test result reporting: Test results can be delivered directly to the mobile device. This frees doctors from having to go to a specific PC workstation to retrieve test results.
- Medical information: Access to the latest medication formulary, disease description, symptoms, and treatment as well as access to clinical procedures can be provided on a mobile device [10].

Recently, clinical applications have smoothly shifted from PDAs to smartphones and tablets because of their numerous advantages. Smartphones can be used to maintain multiple calendars or contacts at numerous locations (eg, office, home) by synchronization using several methods (by Bluetooth, Wi-Fi, or a USB connection). Many devices now have built-in keyboards that allow for rapid data entry. Almost all new devices have touch screens that allow data to be entered interactively. Memory and processing power are no longer issues: most of them have either adequate internal memory or the ability to expand the data storage by inserting extensions and have multicore processing power. The use of certain typical phone-based features, such as short message service (SMS), can, however, experience very significant lag time or reliability problems and should be considered as not appropriate for critical applications, except if there is a specific infrastructure [14].

## Methods

With the help of a team of experts we have identified the challenges that must be faced in the implementation of a mobile application in health care and illustrated them in the context of an application that manages the daily interventions of nurses.

### Background

The University Hospitals of Geneva (HUG) is a consortium of public hospitals in Geneva, Switzerland. It provides primary, secondary, tertiary, and outpatient care for the whole region with 50,000 inpatients and 950,000 outpatient visits a year. The CIS of the HUG is mostly an in-house developed system. It is a service-oriented and component-based architecture with a message-based middleware. It is written in Java with J2EE and open frameworks.

The CIS of HUG has been developed to access all medical information through personal computers. It allows managing most modern CIS tasks such as e-prescription, clinical pathways, care management, laboratory imaging, etc. Despite all the advantages brought by the use of electronic health records, caregivers have rapidly expressed the need for improved mobility. The deployment of a large number of laptops on wheels as an attempt to solve this problem has only partially improved nurses’ mobility. The situation remains unsatisfactory, but the recent explosion of smartphones offers new opportunities that need to be better exploited.

### The Target Application

The purpose of developing our mobile application is to provide bedside management of nurses’ daily interventions. Interventions cover all type of treatments that can be provided to a patient, such as care, drug administration, counseling, and discussions. An intervention is defined by several parameters such as its type, whether it is floating or it has strict timing, date planning, and start/end dates, etc. Depending on their types, interventions are planned by various care providers, such as physicians or nurses. When a clinician prescribes an intervention such as giving a drug, the drug, delivery, dose, duration, and the frequency of the treatment are defined. Each intervention can then be executed, at some time, some place, and in some context. It can also be partially executed or not at all, or rescheduled. All these actions have to be documented.

There are no global standardized guidelines regarding the way nurses have to manage their daily interventions. While there are a lot of differences in the way nurses work from one country to another, for example because of the legal framework (eg, self drug dispensation is generally country specific) or the working context (eg, ICU), there are some general similarities. Before the introduction of mobile computers, their workflow usually followed a sequence of actions relying on printed lists of interventions. These printouts are used by nurses to follow tasks and record their remarks before reporting everything to the system when possible. This process has only partly changed since all wards have laptops. Indeed, the trolley often stays at patients’ room doors preventing access to the CIS at bedside.

In the HUG, nurses are made aware of their daily interventions through a service of the CIS known as the “interventions manager”. This service allows handling interventions lists in numerous ways such as by shift, by type, by room, by nurse, etc. The interventions list guides nurses during their shifts and indicates the tasks to perform. Every time nurses perform one of these interventions, they have to document how the task has been done. When working without laptops, they have to go back to the desk to input the information in the system.

**Figure 1:**
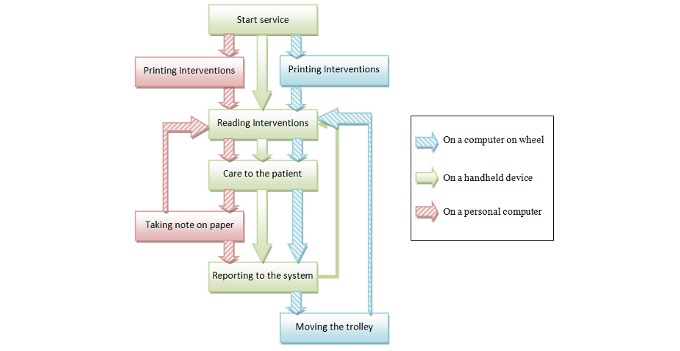
HUG nurses’ workflow according to different devices.

By introducing handheld tools, we can try to suppress all these cumbersome steps to keep the process as simple as possible. There is a strong demand for highly mobile devices that could be carried in a pocket while keeping a decent screen size, such as five inches. Such a device is considered is much better for those using laptops and is expected to replace paper for those still preferring this form. Thus, it is expected that the efficiency of nurses will increase once they are provided with more effective mobile tools (Figure 1), especially in decreasing errors and speeding up the process.

### Development Methodology

The development team consisted of 5 people: a computer scientist, an ergonomist, and 3 domain experts (1 physician and 2 nurses) working in focus groups. The 2 nurses were selected by the Director of Nursing of the HUG (about 4000 nurses). All were research nurses with a strong clinical background and substantial experience in health care and transversal understanding of the problems faced in nursing.

After defining the general architecture of the project, the team went through an iterative process that frequently switched between programming steps and meetings where the prototype graphical user interface (GUI) was tested and discussed (agile methodology). Testing the tool in real working conditions would have been very complicated as it would have implied the connection of our system with the institution’s CIS. It would have required obtaining many authorizations and involved substantial risks to alter the coherence of the clinical data. Consequently, the tool was tested only in a test environment, with predefined care scenarios. The purpose of adopting such an approach for the implementation of the GUI was to emphasize the requirement of a concerted and scientifically grounded approach to develop a GUI. This is still rarely the case, as most GUIs are developed as a result of direct interactions between users and developers, or worse, users and commercial representatives. Most GUIs are also a historical evolution of additive changes.

During the discussions, many interrogations have naturally emerged, not only about the GUI, but also regarding the deployment of such tools. The points discussed were about:

- hardware, form factor, speed, connection, battery autonomy, reliability, maintenance, cost, etc
- architecture, generic mobile device bridge, security, efficiency, etc
- programming languages, portability reusability, ease of finding developers, environments, etc
- privacy, authentication, etc
- ergonomics, user interface; how to have something user-friendly but most of all, prevent increase of acquisition errors
- governance, who pays for the device, is it possible to use one’s own private device, etc

For each of these challenges, the team of experts performed a risk/benefit analysis based on their experiences and on findings from the literature [15]. The group did not proceed to a systematic literature review; however, most papers published these last years have been carefully read to search for methodological approaches that could help the introduction of handheld devices in bedside care.

## Results

The introduction of mobile devices into the care workflow implies dealing with many constraints. The workflow of caregivers must be modified to benefit from the advantages of the new platform. Financial resources must be freed up for the acquisition of the material. Moreover, specific constraints related to mobile environment must be handled with care. Mobile platforms have limited power; they access information through wireless local area networks (WLAN) and have a much smaller screen than any personal computers. All these constraints are emphasized by those related to the health care environment. In such environments, data must be handled with special care. The life of a patient may depend on the integrity and availability of the data.

All these constraints have been regrouped under five different challenges concerning the required financial resource, the hardware, the architecture, the security, and the interface.

### Dealing With Limited Financial Resources

#### Challenge

In the long term, technological changes can lead to substantial financial benefits. However, these changes often require a strong initial investment. The introduction of mobile devices in a care facility is no exception. In the transition from personal computer to mobile device, important costs are incurred.

- The cost of the device: Even if the computational power of the device has increased, their costs have not dropped significantly.
- The cost of the development: The applications running on a personal computer must be adapted for the new platform.
- The cost of the training: Using a new tool induce a change in the workflow of the user and to learn the optimal way to employ the new tool.

#### Solution

As mobile devices become more and more widespread among the general population, it is likely that in a few years, everybody will be equipped with their own device. According to this hypothesis, caregivers could use their personal devices to host clinical applications. Bringing caregivers’ own devices into the hospital has many implications that should not be underestimated. Technically, the development should be multiplatform and allow complete encapsulation of the code and the data. The different display sizes should be taken in account to insure that the developed interface can scale properly. Regarding the security, there are some serious concerns about the applications installed by users that can possibly transfer sensitive information to a third party. Finally, there is concern about the workflow interruptions that can happen frequently when users receive personal notifications on their devices.

#### Example

Being able to run a program on every device on the market is obviously a prerequisite to use the heterogeneous devices owned by caregivers. To make it possible, one solution is to use multiplatform languages, such as Flex or HTML 5, or to be able to compile/translate applications from one operating system to another.

### Choosing Appropriate Hardware

#### Challenge

The choice of mobile device is very important as it constrains or facilitates visibility, usability, and dictates the available computational power. For instance, a large size device (10-inch screen) offers good visibility and allows the display of different information at the same time. However, it is difficult to hold with one hand and impossible to carry in a pocket due to its weight and size; it forces caregivers to put the device on the patient’s bed or table during care. On the other hand, a small device (4-inch screen) can be manipulated easily with one hand and held comfortably in a pocket. However, it provides a limited area to display information [16,17].

#### Solution

Due to the numerous mobile devices available in the market, it is difficult to identify the best device for the clinical environment. Each device possesses their own advantages and drawbacks, and not one stands out in the crowd. With our focus groups, we defined the following criteria:

- Best hand ergonomics: Every user should be able to carry the device in one hand.
- Pocket: When providing care, there must be a way to put the device in the pocket, as there is no good other place to put it.
- Maximum screen size: On small screens, the necessity for scrolling can be a source of problems as the information that is not directly displayed on the screen can be easily missed by the user.
- Best screen resolution: Screen resolution is another parameter that will influence the amount of information that can be displayed on the screen and thus influence the scrolling.
- Lowest weight: Carrying a heavy device all day long is cumbersome.
- Longest battery life: The device’s battery must last at least long enough to perform all the daily tasks of a user.

#### Example

**Figure 2:**
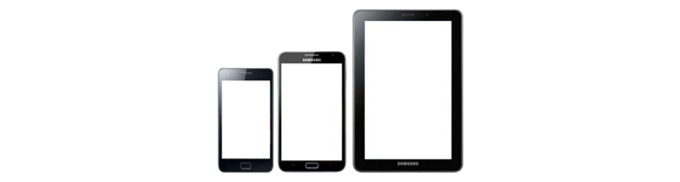
Comparison of device sizes (GALAXY S, GALAXY Note and GALAXY Tablet).

In June 2012, many devices were available on the market all with their own characteristics. As it had been a cumbersome process to compare all of them, we decided to perform the comparison on three devices each representative of a different format (Table 1). The devices selected at the time of this work were (1) the common mobile phone device, the Samsung GALAXY S (Figure 2, left), (2) an intermediate format with the Samsung GALAXY Note (Figure 2, middle), and (3) the tablet format with the Samsung GALAXY Tab 7.0 (Figure 2, right).

**Table 1:** Comparison of three different devices.

Model	Inches	Height	Width	Resolution	Weight	Screen
GALAXY S	4	122.4 mm	64.2 mm	233 ppi	119 g	480 x 800
GALAXY Note	5.3	146.9 mm	83 mm	285 ppi	178 g	800 x 1280
GALAXY Tab 7.0	7	193.7 mm	122.4 mm	170 ppi	384 g	600 x 1024

The unique format of Samsung GALAXY Note has played a crucial role in its adoption by the focus group. Its display is significantly larger than most smartphones, but it remains small enough to be kept in one hand and stored in a large pocket such as in nurses’ and doctors’ garments [18].

### Sustainability

#### Challenge

One of the most disruptive paradigmatic changes that has occurred with the explosion of smartphone is the “App”. The concept of applications has brought many great changes, such as ease of installation, freedom of choice, explosion of products. It has also brought some problems, such as quality assessment and, most of all, the end of sustainability. Whereas the extremely short life cycle and life expectancy of apps are nice for many aspects, it is a potential problem in the clinical informatics: There is a need for a quality software, clear liability, strong life cycle with backwards compatibility, etc.

#### Solution

There is no clear solution, except a set of rules mostly for the governance level. For example, the choice of a programming language that is sustainable and supports nonregression tests, teams, and code management, such as Java.

### Linking the Mobile Device With the Existing Clinical Information System

#### Challenge

Mobile devices must be connected with the CIS to access clinical information. It is mandatory to remain independent of any legacy system to insure easy evolution and effortless maintenance, both for the CIS and the devices used.

#### Solution

The most promising approach is to define a generic bidirectional bridge. The definition of a bidirectional gateway server provides centralized access to any required information between the mobile application and the CIS. Thus, integrating any mobile application requires only integrating this bridge. In addition, the bridge separates the services that are available remotely from the ones proposed as normal Web services. The gateway server is responsible for formatting the data properly before sending it to the appropriate application on the device. Once the mobile device receives the data, its embedded software is responsible for displaying the data through its interface and allows the interaction with the user.

#### Example

**Figure 3:**
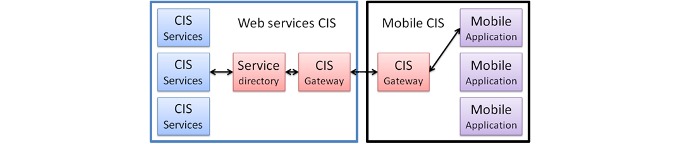
Communication architecture between mobile applications and existing CIS.

Figure 3 shows the link between our mobile application and the current CIS. The CIS of our organization is a component-based architecture, is services and message oriented, with full Java and J2EE. A specific “bridge” component (CIS gateway) has been built to ease communication with mobile devices, providing secure access to data structures and potentially using specific features such has geolocalization. This bridge is generic. It allows also the transport of a description of the interface that can be entirely dynamically built. When a mobile application requires data from the CIS, it communicates with the mobile gateway that transmits the request to the CIS gateway. The service directory is then queried to identify the appropriate service where the required information can be retrieved. The information then returns through the same channel. All data transiting through the channel are formatted in XML [18].

### Data Protection and Authentication

#### Challenge

Data security is crucial in a health care environment. The diffusion of medical information about a patient can have disastrous consequences on his/her life. Thus, correct authentication of authorized users on mobile devices to ensure appropriate data access is a central issue. Common strong authentication policies on desktop computers such as a one-time password, challenges, and pin card are not adapted for mobile devices. Indeed, mobile devices require frequent authentication as they often lock themselves automatically when not in use for a short period. Therefore the chosen authentication method must be free from cumbersome manipulations.

Another risk related to the use of mobile device is theft. This risk is especially strong in a semipublic environment like a hospital where no physical access restriction is applied in most areas. Whereas personal computers are difficult to steal due to their size and the fact that they can be easily secured, a mobile device can easily be stolen or lost. A theft would result in loss of information and confidentiality. In such cases, the access to patient data could have much more serious consequences than unauthorized access to a corporate network [19-21].

#### Solution

As an alternative solution to external hardware, such as the pin card, it is possible to use built-in mobile features, such as embedded cameras or graphical patterns, to authenticate users. The presence of cameras on most mobile devices could allow us to leverage facial recognition [22]. On the other hand, as there is no simple way to prevent a theft, no information about the patient can remain on the device in case the mobile device is stolen. That is, all patient-related information is only continuously stored on the server side or strongly encapsulated on the client side.

#### Example

In the current CIS deployed at HUG, when a user logs in on a personal computer, login requires a “Smartcard” with a pin code. Unfortunately, the use of such authentication methods cannot be applied to mobile devices due to the practical impossibility of linking a smartphone to a card reader. Instead, we investigated face recognition, which some new generation devices with good processing power offer in real-time. The authentication is almost immediate when looking at the device and requires no specific manipulations. In order to minimize the problem in case of theft, data are not stored locally, but the devices are regularly synchronized to store original data on the central server. However, a sufficient amount of information must remain, encapsulated on the client volatile memory, in order to provide availability on the local device in the case of a network crash.

### Designing an Effective Interface

#### Challenge

The proper implementation of user interfaces and interaction models in clinical contexts is often underestimated. The lack of visibility of some information can easily lead to errors and jeopardize the health of the patient. This problem is accentuated when working with mobile devices. There is little research analyzing the impacts of these new interfaces, such as using eye tracking to evaluate the screen exploration or evaluating cognitive load and cognitive tunneling.

The transition of a program developed for personal computers to mobile devices is not as simple as performing a downsizing of the desktop interface to fit the mobile device screen. Indeed, the unique characteristics of mobile devices often require an entire rebuild of the existing interface. Regardless of performance offered by the technology, the usability of mobile information services consequently suffers from interfaces being very compact and cluttered with information and use thus demanding the user’s full attention. In mobile use contexts (eg, finding one’s way through a building, listening to a conversation), the constant change of focus from activities in the real world towards operating technology can be problematic. In order to insure a high usability while actually being mobile, the user interface must remain relatively simple and minimizes the required interactions [23,24]. The selection of the pertinent information to be shown or acquired becomes a major objective.

#### Solutions

In order to build a usable and useful mobile interface, five main principles should be respected.

##### Homogeneity

This first principle recommends keeping a familiar and homogeneous interface. It takes much more time to train users for an application with an interface designed completely differently from a former known version. Giving an interface unfamiliar features, colors, and figures is not appreciated and makes the working process more prone to human errors. Applications with a familiar design increases user acceptance as well as security [17].

##### Hierarchical Organization

Hierarchical organization is a good way to deal with the problem of small displays, especially because it still remains difficult for systems to have an a priori selection of what will be pertinent. The hierarchical organization of information allows the users to increase granularity as needed. It is always possible to increase the depth of hierarchies with additional regroupings. However, it is also important to minimize the learning curve that is associated with the complexity of the hierarchy and to keep the user’s interactions as simple and fast as possible. Therefore, hierarchy must be handled with care as the deeper the hierarchy, the more interactions required by the user are numerous [25,26].

##### Dynamic Organization

Even with the hierarchical organization, all the items cannot be displayed on the screen at once. Some important elements can be hidden to users and require actions such as scrolling to be shown. In order to minimize the risk of missing important information and to minimize the need for user’s interactions, we can capitalize on the real-time usage of the devices. The dynamic organization of the data can optimize the information shown at any time according to the actions to be performed, such as nursing interventions in our case.

##### Context Awareness

It is possible to present only information relevant to a specific situation by making mobile computer systems aware of the user’s contextual setting [27-29]. The idea of context awareness is based on the user’s situation and context, so that the information already provided by the context becomes implicit and does not need to be displayed. Hence, the user’s environment becomes part of the interface

##### Indexicality

The last principle relies on semiotics theory to advise the use of contextual information to improve the user experience. Semiotics concerns the meaning and use of signs and symbols. From a semiotic perspective, information is viewed as representations of something else (their object). Faced with an interpreter, these representations cause a reaction or interpretation. The semiotics operates with three types of representations: symbolic (conventional), iconic (similarity), and indexical (material/causal). Symbols and icons are ways of representing information independent of context, eg, text and graphical illustrations. Indexical signs, on the other hand, are ways of representing information with a strong relation to something else. Indexical representations are, eg, used on signposts and information boards [30].

#### Example

##### Homogeneity

The mobile device being used with an existing CIS, some characteristics of the existing interfaces, such as naming and color charts, are reused in order to build an impression of “déjà-vu”. This can be achieved while exploiting at their best the new paradigms of mobile devices.

**Figure 4:**
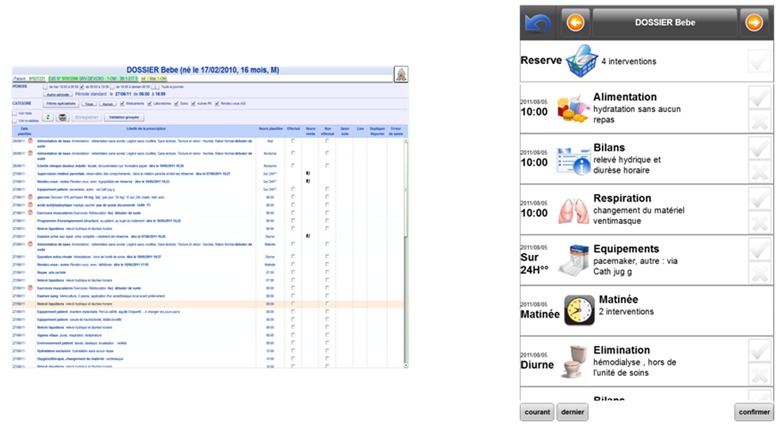
Intervention management interface on a computer screen in the HUG CIS and on the mobile device application.

Figure 4 shows the intervention management interface of the HUG CIS on a personal computer. Each line represents a single intervention. An intervention is described by its date, description, and execution time. The height of the screen allows displaying almost 30 interventions at once. Moreover, the sufficient width permits the display of the full description of the intervention on a single line. To continue with a familiar display on the mobile phone, we have kept this overall organization and respected the naming of elements while keeping only the most significant semantic content and a global chronologic sorting.

##### Hierarchical Organization

**Figure 5:**
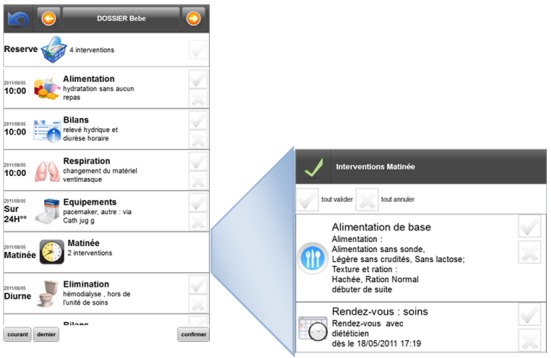
Expansion of an hierarchical item of the mobile interface.

The hierarchical organization has been performed as follows:

1. Interventions of a similar top level are regrouped in a common item. Based on the hypothesis that tasks of a similar type are usually performed at the same time by the care provider, all the interventions happening at a similar time are regrouped, if they share a similar top-level type. For instance, if a nurse must dispense several drugs at the same time, they can be regrouped under a single task with various actions. Regrouping the interventions of a similar type not only helps organize the work in a clever way but also offers a much better overview of the tasks to perform (Figure 5Error: Reference source not found).
2. Nonscheduled interventions are regrouped in a single group. Nonscheduled interventions, such as PRN (Pro re nata) drug orders, are not compulsory but are available according to certain situations, in a given frame. For example, there may be some pain treatment held in reserve for the patient. These are “floating” actions, possible until they are made and that have to follow strict rules, such as a maximal dose per 24h. As these interventions remain always available, displaying them in the main screen can take all the space available. To avoid this, all interventions in reserve are regrouped in a single item that remains always at the top of the list and only as long as they have not been completely used. This ensures improved visibility at any time.

**Figure 6:**
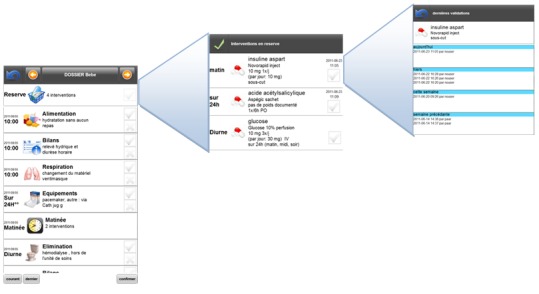
Hierarchical navigation through PRN drugs.

When the user selects such groups, the contextual information is displayed (Figure 6). If actions are required, they can be directly entered in this contextual frame, such as in the example displayed. If an alert or decision support is available, it is also directly shown at the right place.

##### Dynamic Organization

The dynamic organization has been performed as follows:

1. By default, the first displayed intervention is the one to be performed at the current time: The real-time usage of the mobile device allows focusing the display on the intervention to perform at the exact time the device is used. In case older interventions remain invalidated, nurses still have the possibility to return to the older interventions that must be validated. This strategy minimizes the number of manipulations required by the user and saves precious time while focusing on relevant information.
2. The interventions valid over a range are ordered dynamically: Whereas the ranking of interventions scheduled at a precise time is logical, there are no clear rules about the interventions to be executed in a period of time, such as “in the morning”. After several iterations, the solution implemented is to regroup all interventions of periods covering the current time in the next 1 hour period. Therefore, these interventions will slide in time all day as long as they are valid and not yet completed. For example, at 3AM, any intervention scheduled in the “morning” period, according to its definition for this ward, will be shown in the 4AM frame. At 4AM, they will slide to 5AM, and so on, as long as the time is in the period, and the interventions must or can be executed. This solution has been chosen to minimize the risk of missing the intervention.

##### Context Awareness

**Figure 7:**
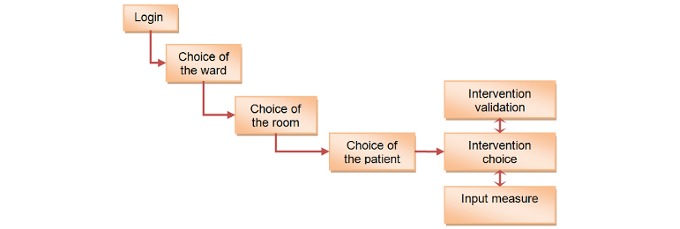
Steps to select the relevant interventions.

Every nurse followed a succession of steps ( Figure 7) to define the working context: (1) Choice of ward: if the nurse has access to more than one ward, it is possible to select the one for the daily tasks (2) Choice of room: In large wards, nurses are not responsible for every room; therefore, nurses can select the rooms containing patients they have to visit, and (3) Choice of the patients: Once the rooms have been selected, nurses can choose the patient to start work with. Afterwards, they can switch from one patient to another directly in the intervention view.

Once a patient has been selected, nurses get access to all the patient interventions. This list of interventions is ordered and displays tasks to perform at a given time according to rules mentioned above.

**Figure 8:**
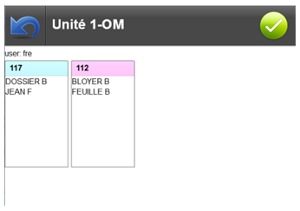
Screen for the selection of the rooms in the care unit.

**Figure 9:**
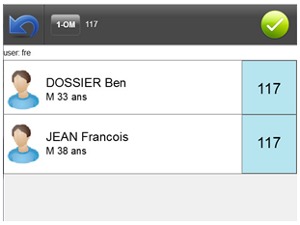
Screen for the selection of the patients in the rooms.

Figure 8 shows a screenshot of the interface displayed to choose the rooms in the selected care unit. Each room is represented by a panel with the number of the room as title. The names of the patients are displayed inside the panel. Once one or several rooms have been selected, nurses get to a screen similar to the one in Figure 9. On this screen, every patient occupying one of the selected rooms is displayed. Patients are presented with their picture, if available, and some demographics.

**Figure 10:**
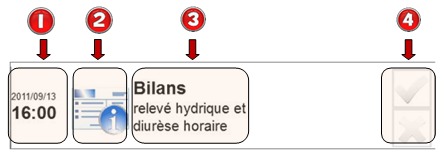
Indexicality indicators of every intervention item.

##### Indexicality

In our development, every item relies on semiotics to improve visibility, simplify information retrieval, and thus decrease the learning curve and errors (Figure 10). There is an icon representing the task to be performed and a temporal indication showing the date and time of validation. This representation allows users to locate easily the task to perform, as they are organized in a logical order. Moreover, the task is clearly identifiable by simply viewing the iconic representation and helps give an overall view of the tasks to perform.

## Discussion

In this study, we evaluated the potential problems related to the deployment of a mobile clinical application and tried to find solutions based on scientifically validated evidences. This approach was motivated following the unexpected increase in acquisition errors observed in using mobile devices.

### Define a Clear Strategy

A clear strategy must be defined for the institution, taking into consideration all aspects of the deployment. The choice to buy and deploy devices involves finding out which platform is most suited for maintenance, sustainability, fast and large automatic deployment of applications, etc. The same questions go for the software: how will it be maintained and deployed, if it is an app running on the client side, etc. Maintenance, sustainability, costs, authentication, data safety, learning curve, and acquisition errors among others are all important aspects.

### Do Not Underestimate the Hardware

Choosing appropriate hardware is rarely taken seriously in a domain that is mostly market driven. However, it is an important step, and not only for technical reasons. There is a long list of elements such as autonomy, device hygiene, and device size that can be evaluated, according to needs, context of use, local IT culture, etc. For example, despite initial enthusiasm, the experience we had with tablets for nurses was not successful after a few months simply because these tablets did not fit in the pockets of professional garments.

### Generic Interoperability Framework

Technical and semantic interoperability is probably one of the most important challenges of the field of biomedical informatics. In nomadic computing, which is an emerging technology, there should be efforts made to start with a clear and coherent, semantically oriented framework from the start, such as openEHR, CEN 13606, or RDF. In the future, more formalized data formats such as those employed in the SMArt project [31] can be adopted to facilitate the link between the mobile platform and any CIS.

### Safety of Data

Data security is crucial in a health care environment. The diffusion of medical information about a patient can have consequences, for the patient and for the organization’s image and liability. Data integrity is also crucial, especially in making sure that no data are lost, data are stored and retrieved in a proper manner, that there is a coherent management of concurrent editing, etc. This has a huge influence on nomadic devices, especially when deciding if an intermittent connection is supported [19].

### New Human-Machine Interaction Paradigms

Small screens have a huge influence on information organization, display and retrieval. Jones et al [32] performed an experiment where users had to find information on the Internet using two types of screens. They report that users of the small screen answered half as many questions correctly as the large screen group. Moreover, 80% of users of small screens indicated that they felt screen size impacted on their ability to complete the tasks, compared to 40% for large screen users. The increased amount of scrolling needed plays a large part in both degrading speed and retrieval performance [33]. Marsden, Cherry, and Haefele [34] found that users often do not scroll down on a page because they simply did not see the scrollbar and were unaware that more information was available. If users do not scroll down, options on the first few lines of a display will be selected faster than options further down in a list, even if such an option is not correct. Scrolling and paging also more often results in errors because users try to select an option from the visible options instead of scrolling down to the end or looking at more than one page. This is a complication that might lead to the preference of narrower hierarchies on smaller screens, to prevent users from scrolling unnecessarily [35].

### Conclusion

Working in a clinical environment requires mobility and constant access to clinical information. The necessity for switching continuously from paper to computer, or to carry a laptop at all times, or to walk back to offices to access computers, creates an enormous amount of work and represents a source of errors. With the maturity of personal mobile assistants such as tablets and smartphones, it is now possible to imagine a fully integrated tool to access and manage clinical information anywhere, anytime in the hospital. However, the deployment of a clinical application on mobile platform is not a simple task. Unexpected side effects have been described in the literature, one of the most important being decreased perception of the information and increased errors during data acquisition. This paper identifies some of the challenges raised when using such devices. Currently, there is a lack of clear evidence identifying the risks, benefits, and solutions related to the various contexts for using these devices.

## Abbreviations

CIS: Clinical Information System
GUI: Graphical User Interface
HUG: University Hospitals of Geneva
PDA: Personal Digital Assistant
WLAN: Wireless Local Area Networks
XML: Extensible Markup Language
RDF: Resource Description Framework
SMArt: Substitutable Medical Applications, reusable technologies
